# Notes on Sciatica and Lumbago

**Published:** 1905-02-11

**Authors:** 


### NOTES ON SCIATICA AND LUMBAGO.

Dr. J. Michell Clarke[1] has found the Weir-Mitchell method of treatment by means of the long splint very useful in obstinate cases of sciatica. The milder forms of the disease, no doubt, readily yield to ordinary constitutional and local measures, but many severe cases drag on for months and years, crippling the patient and causing much suffering. To be successful the Weir-Mitchell method must receive careful and exact attention. The limb is first carefully bandaged in its whole length with a firm flannel bandage. Then a long splint, which will prevent movement both at the hip and the knee, is applied, and is secured by bandages. The knee should be in a position of slight flexion, and the heel carefully supported. After five days, or earlier if there is definite discomfort, the bandages are taken off and afterwards are reapplied. This, after the sixth day, is repeated daily, when gentle passive movements of the joints are practised. When pain has been absent for several days, generally, that is, at the end of two or three weeks or later, perhaps, in a very bad case, the splint is left off during the day and is reapplied at night. Later it is discontinued altogether, the limb, however, being kept bandaged, and after another week or less the patient is allowed to get up. It is the absence of pain, which is the deciding point in determining the disuse of the splint, and as soon as this can be adopted, massage should be instituted with a view to promoting muscular nutrition. A practical hint is that when the patient first leaves his bed he should be permitted to stand up or to lie down, but should not be allowed to sit. Of 12 patients treated on the above plan by Dr. Michell Clarke the average duration of the disease before coming under treatment was 23·6 weeks (maximum 21 months, minimum 7 weeks); the average duration of treatment by the Weir-Mitchell method was 5½ weeks. Ten of the patients were cured, the exception being a restless, impatient man who would not allow the splint to be continued. One difficulty is to induce patients to submit to the splint during the first 24 to 48 hours; it proves irksome, and sometimes there is sufficient pain to justify the administration of morphine. In neurotic patients there is apt to be a good deal of flatulent dyspepsia, and thus the diet may need careful attention. A detail of importance is the use of a flat bed with the support of a firm mattress, and the complete immobility of the limb is absolutely essential to success. The after-treatment includes massage, tonics, and cod liver oil. Dr. J. Farquhar[2] has found pilocarpine nitrate of value in the treatment of intractable cases of sciatica. He injects it subcutaneously over painful points, using one-third of a grain, every night or every second night, and gives in addition a minim of croton oil followed by a saline purge once a week. The treatment is not advisable where there is organic cardiac disease. Sir Wm. Gowers,[3] in an interesting lecture on lumbago, argues that the disease is a rheumatic and inflammatory affection of the fibrous tissue (fibrositis) of the lumbar muscles. The condition may spread to—sometimes indeed it commences in—the tendinous structures which attach the muscles to the sacrum and the iliac crest. Thence it may also reach the sheath of the sciatic nerve, and even lead to a definite sciatic neuritis. The essential feature of lumbago is this fibrositis which is the condition existing also in other forms of muscular rheumatism; and one of the clinical features of muscular rheumatism is the fact that the pain is not spontaneous as in neuralgia, but is always induced, as by movement. One variety, apt to be confused with neuritis, occurs in the upper limb. It is characterised by extreme pain caused by any contraction of the muscles; and hence even passive movement is painful, as the patient unconsciously throws opposing muscles into contraction. But if when his attention is distracted gentle attempts are made to move the joints, this will be found to be possible, and thus the condition is distinguished from adhesions fixing the joints, which has sometimes in error been advanced as a diagnosis.

There are two measures in the treatment of lumbago and other forms of rheumatic fibrositis the value of which is not fully appreciated. These are diaphoresis and rest. The former is mainly beneficial when applied at the very outset of the attack, and a Turkish bath is perhaps the easiest method of causing the free sweating which is to be desired. But even the lapse of a few days may mean that the sweating will produce little or no good result. Rest is the principal demand of the painful muscles, and when the fibrositis affects the upper limb a sling and complete rest are essential. Hot fomentations and packs are good at the outset, but massage and electricity should be postponed until tenderness has disappeared; not even passive motion should be practised so long as it causes pain. The salicylates seem to have much less influence on rheumatic fibrositis than they have on articular rheumatism, but they may be tried, and salicylate of potassium or lithium is perhaps the most useful form. A useful combination in the early stage is potassium citrate, nitrous ether, and colchicum to which small doses of perchloride of mercury may be added if the attack is one of considerable intensity. The brachial form of the disease is apt to be peculiarly obstinate: it is benefited by superheated air, but the applications have to be continued for weeks. A valuable local measure is the deep hypodermic injection of cocaine repeated daily for two or three weeks, and counter-irritation, more particularly the actual cautery, usually lessens the pain.

An interesting contrast to generally received views is provided by Mr. Jonathan Hutchinson,[4] who asserts that the site of pain in lumbago is not in the lumbar muscles, nor, indeed, in the loins at all. It is to the sacral region that the sufferer refers the pain, and lumbago might be defined as a "liability to pain across the sacrum or in the sacro-iliac synchondroses on movement." The structures involved are the ligaments of the sacrum and the cartilage between the sacrum and iliac bones, and the condition is one of a large group of phenomena which are associated with the inheritance of a gouty tendency and with exposure to the exciting causes of rheumatism. The curative measures, according to Mr. Hutchinson, are active exercise, care in diet, and warm clothing, especially across the hips. During the presence of the pain the sufferer should not rest, but should make every effort to move about. Even though at first he should need support and assistance, he will, if sufficiently resolute, find the pain subsiding and the power of free locomotion returning. If he will not pursue the more heroic course, cupping (which was Graves's favourite remedy), sinapisms, or heat should be applied, and for internal treatment alkalies, aconite, and colchicum should be ordered. Hot weak tea in large quantities is of considerable value both in lumbago and sciatica, and, indeed, in all forms of rheumatic gout. This being Mr. Hutchinson's teaching, it will be observed that his difference from such a view as is announced in Sir Wm. Gowers' lecture is no mere academic one. On the contrary, it enters the practical field of treatment, where it advises a course of conduct which other schemes condemn. The difference of opinion has at least this compensation, that it provides alternate forms of treatment which each practitioner may test for himself.

[1] Lancet, Oct. 17, 1903. [2] Brit. Med. Jour., April 16, 1904. [3] Ibid, Jan. 26, 1904. [4] Archives of Surgery, vol. viii., p. 97.

